# Two Cases of Phthisis Treated by Means of Subcutaneous and Intrapulmonary Injections of Carbolate of Camphor

**Published:** 1888-09

**Authors:** R. Shingleton Smith

**Affiliations:** Senior Physician to the Bristol Royal Infirmary


					TWO CASES OF PHTHISIS TREATED BY
MEANS OF SUBCUTANEOUS AND INTRA-
PULMONARY INJECTIONS OF CARBOLATE
OF CAMPHOR.
Bv R. Shingleton Smith, M.D.,
B.Sc., F.R.C.P., Senior Physician to the Bristol
Royal Infirmary.
A saturated solution of camphor in strong carbolic
acid has long been known; but has attracted little notice
as a therapeutic agent.
Dr. Cochran,* of Shomond, Wis., has recommended
its use, both locally and internally, as an antiseptic agent.
He gives it internally in ten-drop doses in capsule, and
injects it hypodermically; he also employs it externally.
The camphor is dissolved in a 95 per cent, solution of
carbolic acid to saturation ; the carbolic acid dissolves
about three times its weight of the camphor, the resultant
Therapeutic Gazette, December, 1887.
14
Vol. vi. No> 21.
l86 TWO CASES OF PHTHISIS.
being a thin, clear, oleaginous mixture, having a strong
odour of camphor and a faint one of carbolic acid. It
has a pungent flavour of camphor, but no flavour of the
acid; it causes smarting on being applied to cutaneous
abrasions, and when injected under the skin causes slight
stinging for a moment, which then subsides, with little
evidence of local irritation.
The mixture appears to resemble the solution of
carbolic acid in olive oil in this respect, that the irritating
effect of the acid is entirely destroyed by the oil; and it may
be assumed that the antiseptic action is correspondingly
diluted in proportion to the strength. Now, it is stated
by Lauder Brunton* that the strength of disinfectant
required to hinder or promote the development of anthrax
bacilli is about the same for camphor as for carbolic acid,
one in 2,500 being sufficient. Accordingly, it may be
assumed that the antiseptic power of the carbolic acid
is not destroyed or diluted by the intermixture with
camphor, whilst the mixture has no longer any of the
ordinary caustic properties of the undiluted acid.
The solution appears, therefore, to be well suited for
internal use as a germicide in phthisis; and with this
view it has been employed by me in a series of cases, of
which the two following are illustrations.
Charlotte C., set. 20, single, laundress. Had enteric
fever and rheumatic fever eight years ago, and several
attacks of rheumatism since. Mother is a cripple, and
dropsical; has lost one brother in decline, and one with
dropsy after scarlet fever. Patient was admitted to
Bristol Royal Infirmary on February 18th, 1888, having
caught cold about Christmas, when she had a bad
cough and lost her voice. She also complained of chest
Pharmacology, p. 77.
TWO CASES OF PHTHISIS. 187
pains, worse after food, with occasional vomiting; and
had been getting thin. There was no history of hae-
moptysis. She was fairly well nourished, but with
clubbed finger-tips, bluish conjunctivae, and husky, feeble
voice, with a troublesome cough. Her weight was 7 st.
7lbs., and her temperature 102?. There was much gastric
irritability, and some epigastric tenderness. Decided
dulness at right apex, with crepitation, and exaggerated
voice-sounds. Some slight doubtful dulness at the left
apex. Heart-sounds normal. Urine healthy. Was
ordered mixture with bismuth and morphia.
February 22nd. Much sweating at night, and trouble-
some cough, for which atropine and morphia pill was given.
On laryngoscopic examination, the vocal cords were
found to be healthy in appearance, but did not close the
glottis completely: this defect was at once remedied by
the application of the induced current internally. The
sputum was found to contain numerous tubercle-bacilli.
March 2nd. A hypodermic injection (rrtvij.) of the
camphor carbolate was given in the right forearm, and
Was not followed by any indications of irritation.
March 4th. An injection of camphor carbolate with
iodoform (111.x. of one part in five). This was followed
by
a rise in temperature of one degree in the evening,
and some irritation of cellular tissue on day following.
March 10th. An injection of the camphor carbolate
without iodoform was now given daily, ten minims being
introduced on the outer side of one or other thigh.
These injections gave little pain, and did not irritate.
Accordingly, on March 17th, an injection of ten minims
was given in the apex of the right lung. There was no
coughing, no haemorrhage, nor any evidence of lung
irritation.
14 *
l88 TWO CASES OF PHTHISIS.
These injections, intra-pulmonary, below the clavicle,
were repeated on the 20th and 22nd,. ten minims each,
and a similar dose given hypodermically on all other
days.
March 21st. Some induration and swelling of left
thigh followed the injection of previous day; and on
March 23rd a few rales were heard at the right apex,
following the injection into lung on the day before.
March 27th. Another dose of ten minims was injected
into the right axilla, and gave little pain. Some irrita-
tion had been caused by an injection given in the left
arm a few days ago.
April 10th. An injection of carbolate of camphor
with creasote (one in ten) was given in left forearm, and
repeated daily.
On 15th, given over the shoulder, some irritation fol-
lowed, which soon subsided. Patient was taking creasote
(nxj.) three times a day, and cod liver oil with iodoform.
April 22nd. Another injection into right apex?solution
without creasote?as being the less irritating to cellular
tissue. There was no coughing; but patient felt a little
pain on breathing deeply.
April 28th. Another injection into apex of lung gave
some little pain, passing off in a few minutes. No
coughing.
May 1st. Injection into second right interspace gave
rise to no irritation; and on the 3rd the daily dose hypo-
dermically was increased to twenty minims.
gth. An injection of ten minims into the right lung
caused much coughing, and sputum and breath afterwards
had the odour of the fluid injected. No further injections
were made into lung; and on the 13th the daily hypo-
dermic ones were discontinued.
TWO CASES OF PHTHISIS. 189
On April 22nd, occasional rales could be heard at the
right apex, and there was some dulness; but the tempera-
ture was normal: there were no active symptoms, little
cough, no expectoration, appetite good, weight increased.
June nth. Physical signs indicated some little con-
densation at right apex, but there were no active symptoms,
and no sputum could be obtained.
Sept. 10th. General health good. No evidences of
cavity or breaking down in the lung: nothing more than
slight dulness on percussion at the right apex.
The following is a summary of the temperatures,
weight, and injections :
date. temp. weight. injections.
1888. Morn. Even. st. lb.
101 99 77
98.4 99 76
Feby. 18
>> 26
March 1
April
4
10
13
14
15
16
18
19
20
21
22
23
24
25
26
27
31
98.4 IOO ?
98 99 ? Camph. carbolat. ni. vij.?Arm
with iodoform?Leg
98 100 7 6
98 100 ?
98 99 ?
98 98.4 7 7
98 97.6 ?
98 98.4 ?
98 98.6 ?
98.4 99 ?
98 99 ?
98 98.4 ?
98 98.8 7 7
97.8 98 ?
98 98.4 ?
98 98.4 ?
98 99.2 ?
98 IOO
98.4 99.2 ?
98.4 99.2 7 7
98 98.2 ?
99 100.4 ?
98.4 100 7 7
98.6 99 78
Daily
itl x.? Thigh
Apex, right lung, iti.x.
itl x.?Thigh.
Right lung
Thigh
Right lung
Thigh
Lung, axilla
Hypodermic in thigh
190 TWO CASES OF PHTHISIS.
April 10
.. 13
? 14
.. 15
>. 16
.. 17
24
? 28
? 29
30
May 1
3
4
5
6
7
9
? 10
12
.. 13
? 14
>> 21
Sept. 10
WEIGHT. INJECTIONS.
Even. st. Ib.
99 ? ... Camph.carbolat.
Daily with creasote?Thigh
? 1, ? 111.x.(iinio) ,,
90 99.2 ?
97.6 98.4 ?
98 98.4 ?
98 99 7 8
97-4 98 ?
97.4 98 ?
97.4 98.2 7 10
97.4 98 ?
? 97-2 ?
? 98.2 7 11
? 98.2 ?
? 98.2 ?
? 98.2 ?
? 98.6 ?
? 97.6 ?
? 97.4 7 12
99
99.2
99
? ,, ,, L. shoulder
? >. ,, ? L. forearm
? >> ,, ,, R. forearm
Daily injections discontinued.
Camph. carbolat., in.x.?Apex right
[lung
? Right apex
? Arm
Thigh
2nd right interspace, rrt x.
Right arm, in. xx.
R. supra-spinous, in. xviij.
Forearm, in. xx.
Buttock, 111 xx.
Right apex, ttl
Buttock, it], xv.
rn. xx.
Arm, in. xx.
100 7 12 ... Injections discontinued.
8 o
8 4
It will be seen that about fifty injections in all were
made in the course of ten weeks ; of these nine were into
different spots of the condensed right upper lobe, whilst
the remaining forty-one were into various parts of the
subcutaneous cellular tissue. The average quantity in-
jected amounted to about ten minims, so that the whole
quantity injected in the course of ten weeks only slightly
exceeded one ounce. This comparatively small amount
could obviously have no very powerful antiseptic or germi-
cide effect when distributed throughout the blood of the
whole body, so that we must look to the local action of
the drug on the part injected if we are to get any definite
TWO CASES OF PHTHISIS. 191
germicide - result. The subcutaneous injections were
mainly intended to test the characters of the fluid injected,
in order that it might be shown that the fluid to be in-
jected into the lung was not powerfully irritating to the
tissues elsewhere. Variations were made in the fluid on
two occasions. An iodoform solution in camphor carbo-
late (2 grs. in itj, x.) was used once, but gave rise to so
much inflammatory cellulitis that it was not repeated.
A solution of creasote in camphor carbolate (1 in 10) was
injected subcutaneously on six occasions; but this was
followed on each occasion by some slight local irritation,
which subsided after a few days. Neither the iodoform
nor the creasote solution was used for injection into lung.
As regards the immediate effect of the intra-pulmonary
injection, there was little to observe. On only one occa-
sion did any violent coughing occur; but on this occasion
the fluid must have reached a bronchus, for the odour of
it was at once detected in the breath and the expecto-
ration. There was so little discomfort from the intra-
thoracic injections, that the patient herself preferred those
to the subcutaneous ones, "because they did not hurt so
much," and they gave rise to no discomfort afterwards.
A careful examination of the temperature'-chart shows
that on three occasions there was a rise amounting to one
or two degrees on the day after the injection, but there
were no symptoms of pleuritis or any other evidence of
internal irritation.
It is always difficult in a case of phthisis to justly
estimate the value of the treatment adopted, and hence
we can only say that the methods employed appeared to
give an eminently satisfactory result: the general im-
provement in the patient's condition was unquestionable.
The case looked at first like one of acute progressive
192 TWO CASES OF PHTHISIS.
disease; but all active symptoms were arrested : the
catarrhal throat soon got well; the night - sweats and
wasting ceased; the temperature became absolutely
normal; the sputum, which had been crowded with
bacilli, could no longer be obtained for examination;
and the patient considered herself quite well. She at
once resumed her occupation as laundress; has con-
tinued in good health, and is still gaining weight.
Mary P., aet. 18. Previous good health; good family
history; getting thin for two months; cough and cold
two weeks. On night of March nth, during a fit of
coughing, she suddenly brought up about a quart of
bright red blood, and was admitted to Infirmary on
following day.
On admission was fairly well nourished and of good
colour. Finger-tips not markedly clubbed. Pulse quick,
but of good volume. Temperature ioi? in evening.
Dulness at left apex, front and back; expiratory sounds
prolonged, and coarse crepitations, with increased vocal
resonance. Little cough, and no expectoration. Pityriasis
versicolor on chest.
Haemoptysis recurred on 12th and 13th in small
quantity, and the evening temperatures ranged from ioi?
to 103?. On the 18th there was extensive dulness at both
bases, with abundant fine crepitation.
April 3rd. It was noted that breath-sounds on right side
were good; but still crepitation and some dulness at the
left base, and also at the left apex, both supra-spinous and
infra-clavicular.
April 20th. No return of haemoptysis; base of lung
clearing slowly, and no active symptoms. Temperature
had gradually come down to the normal, and weight was
stationary at 7 st. 9 lbs.
TWO CASES OF PHTHISIS. I93
Injections of carbolate of camphor commenced on the
21st; at first ten minims, afterwards increased to twenty:
given first in left thigh, then in right. No irritation
followed; accordingly, on 24th, an injection into left
upper lobe in front was made, 10 minims, giving little
pain or irritation, and no coughing. Temperature rose to
100.20 on day following, but was normal in two days.
Subcutaneous injections were resumed on the 27th, and
an intra-pulmonary one given on the 28th. Daily in-
jections were given under the skin, and a third into left
apex on May 1st, a fourth on May 4th, and a fifth, of 15
minims, on May 8th. They were then continued on
alternate days, and discontinued on the 15th. Eighteen
injections in all were given in about three weeks ; five
into apex of left lung, and thirteen into the subcutaneous
tissue of different parts. One injection gave rise to a
small boil in the thigh; the others were painful for a few
seconds, but gave no discomfort afterwards.
On May 22nd there was still slight dulness at the left
base, also at left supra-spinous region; in front there was
medium-sized crepitation and bronchophony below the
left clavicle. There was little cough, but no expectora-
tion. No sputum had been obtained for examination.
Evening temperature had been normal for two weeks, and
general health was good.
DATE. TEMP. WEIGHT. INJECTIONS.
1888. Morn. Even. st. lb.
Mar. 12
16
>> 20
.. 23
? 30
April 7
,, 14
>> 21
99.6 101
100 103
99.4 101.6 7 9
98.6 101.4
98.6 100
98.6 99-6 7 9
98.6 101.2 7 9
98 98.4 7 9 ... Camph. carbolat., rn. x.?Leg
194 TWO CASES OF PHTHISIS.
TEMP. WEIGHT. INJECTIONS.
Mom. Even. st. lb.
April 24
.. 27
? 28
>, 29
>. 3?
May
3
4
5
6
7
8
10
12
13
15
19
98.6 99.2 7 9
98.6 99 ?
98.4 99.2 ?
98.6 99.6 ?
98.2 98 ?
98.6 99 ?
98.2 99 ?
98.6 99 79
98 98.4 ?
98 98.4 ?
98.2 98 ?
98 98.4 ?
98.2 98.4 7 9
?4
97
Camph. carbolat.
in. x.?Apex left lung
>> >> Left leg
>? >> Left apex, lung
>1 >> Right leg
>> >> Left thigh
) t 1. Left apex, lung
? ,, Left leg, rn. xx.
>> ?> Left apex, lung, itlxv.
,, ? Right thigh, m xx.
,, ,, Left thigh
,, ,, Right thigh
,, ,, Left apex, lung, iti xv.
,, ,, Right gluteus, in. xv.
>. >> >> .. m xx.
>, .> ,> "1 xv.
In this case also the injections gave rise to a satisfac-
tory result. The whole quantity injected was 245 minims,
within twenty-five days, 60 minims having been diffused
through the diseased patch at the apex of the left lung.
On one occasion there was a rise in temperature of two
degrees on the day after injection; but, commonly, there
was no cough, and no evidence of lung-irritation. At no
time could any sputum be obtained for examination; but
the patient had abundant evidence otherwise of the
bacillary nature of her disease. When treatment was
discontinued she was quite well, with no lung-symptoms,
and with normal steady temperature : her disease, which
threatened to be acute and active, appeared to have been
entirely arrested.

				

